# Modeling and Optimization of Gas Sparging-Assisted Bacterial Cultivation Broth Microfiltration by Response Surface Methodology and Genetic Algorithm

**DOI:** 10.3390/membranes11090681

**Published:** 2021-09-01

**Authors:** Aleksandar Jokić, Ivana Pajčin, Nataša Lukić, Vanja Vlajkov, Arpad Kiralj, Selena Dmitrović, Jovana Grahovac

**Affiliations:** Faculty of Technology Novi Sad, University of Novi Sad, Bulevar cara Lazara 1, 21000 Novi Sad, Serbia; jokic@uns.ac.rs (A.J.); nlukic@tf.uns.ac.rs (N.L.); vanja.vlajkov@uns.ac.rs (V.V.); arpadkiralj@uns.ac.rs (A.K.); selena.dmitrovic@uns.ac.rs (S.D.)

**Keywords:** microfiltration, gas sparging, response surface methodology, desirability function, genetic algorithm, permeate flux, specific energy consumption, microbial biopesticide, *Bacillus velezensis*

## Abstract

Production of highly efficient biomass-based microbial biopesticides significantly depends on downstream processing in terms of obtaining as high concentration of viable cells as possible. Microfiltration is one of the recommended operations for microbial biomass separation, but its main limitation is permeate flux decrease due to the membrane fouling. The effect of air sparging as a hydrodynamic technique for improvement of permeate flux during microfiltration of *Bacillus velezensis* cultivation broth was investigated. Modeling of the microfiltration was performed using the response surface methodology, while desirability function approach and genetic algorithm were applied for optimization, i.e., maximization of permeate flux and minimization of specific energy consumption. The results have revealed antagonistic relationship between the investigated dependent variables. The optimized values of superficial feed velocity and transmembrane pressure were close to the mean values of the investigated value ranges (0.68 bar and 0.96 m/s, respectively), while the optimized value of superficial air velocity had a more narrow distribution around 0.25 m/s. The results of this study have revealed a significant improvement of microfiltration performance by applying air sparging, thus this flux improvement method should be further investigated in downstream processing of different bacterial cultivation broths.

## 1. Introduction

Nowadays, conventional agriculture often depends on chemical pesticides and inorganic fertilizers in order to achieve stable and great quantity agricultural output. An environmental concern as a result of the extensive use of agrochemicals has led to development of sustainable agriculture approach [1]. Microbial biopesticides offer an environment-friendly biotechnological alternative to chemical control of plant diseases and pests [2,3]. Utilization of microorganisms or their metabolites in biological control of various plant pathogens represents a fast-growing sector, with expected continued growth in the following years, mostly due to inevitable and necessary development of organic and regenerative agriculture [4,5]. Bacteria of the genus *Bacillus* could be found in the majority of commercially available products for plant protection due to their favorable characteristics for active component of microbial biopesticide [6,7,8]. *Bacillus velezensis* has been investigated in biological control of different plant diseases considering the ability of *Bacillus velezensis* strains to produce antimicrobial compounds and express mechanisms related to plant growth promotion [9,10]. *Bacillus velezensis* IP22 is a novel biocontrol agent, which expresses several mechanisms of antimicrobial action against plant pathogens, including competition for growth space and nutrients, as well as production of lipopeptides [11,12]. Sustainable, environment-friendly, and cost-effective production of biopesticides significantly depends on the method of cultivation broth downstream processing [13]. Cell harvesting, i.e., cultivation broth clarification, is the step in downstream processing with an aim of a solid/liquid separation to recover the microbial cells from their suspending medium in order to increase concentration of the active component in the final biopesticide product. In biotechnological production of biopesticides, it is usually done by centrifugation or microfiltration [14]. The effective recovery of the cultivation broth components, especially microbial biomass considering necessity to maintain cell viability, is the penultimate step in biopesticide production, which can directly affect product formulation and consequently product efficiency. However, when it comes to *Bacillus*-based biopesticides production, this downstream separation step has not been addressed comprehensively yet.

Various membrane separation technologies have been used for processing of fermentation broth of various microorganisms [15]. Cross-flow microfiltration has several advantages over conventional technology for separation of cells from extracellular products in bio-industry [13,16,17]. Alongside many advantages over other clarification methods, a serious drawback of microfiltration is membrane fouling, leading to decrease in permeate flux and consequently making microfiltration process uneconomical [18,19]. Membrane fouling is a result of soluble feed components deposition on the membrane surface as well as the buildup of a compressible layer of rejected biomass (i.e., cake layer). Numerous literature data report that the hydraulic resistance associated with cake build-up is the main influencing factor on micro- and/or ultrafiltration in the presence of microbial biomass [20,21]. Furthermore, molecules present in the cultivation broth could also end up bound in the membrane pores, making membrane cleaning even harder and contributing to significant reduction of its life cycle, which is followed by a continuous permeate flux reduction. Therefore, numerous flux enhancement methods have been developed and experimentally investigated in recent years. Physical or chemical feed-mixture pretreatment together with appropriate choice of membrane material could prevent membrane fouling and contribute to increased permeate flux values, as showed in case of microfiltration of *Bacillus thuringiensis* cultivation broth [22,23]. Different flow manipulations could be applied in order to enhance mass transfer near membrane surface, including application of static turbulence promoters [18,24,25,26] and feed flow alterations, such as pulsing, back-flushing, vibration, etc. [27,28,29,30]. Besides increased microfiltration efficiency in terms of permeate flux improvement, reduction of fouling using the aforementioned methods also contributes to membrane life prolongation [31].

Gas sparging during microfiltration assumes introduction of gas into feed mixture flow and achieving two-phase (gas-liquid) flow which is aimed to cause hydrodynamic instabilities in the membrane channel and thus affect concentration polarization in terms of cake layer removal. The main reason for disturbance of cake layer structure is an increase of turbulence shear rate near the membrane surface, which depends on gas and liquid phase linear speed, but also on the gas-liquid flow regime [32]. The presence of gas bubbles in the membrane channel causes efficient removal of loosely bound macromolecules and particles, while breaking of the cake layer could be caused by gas bubbles bursting or coalescence [33,34]. Gas sparging has proven to be efficient in filtration cake removal, hindering of reversible fouling and consequently permeate flux improvement in the studies including filtration of yeast suspension [34,35,36,37,38,39,40], titanium oxide suspension [41], skim milk [42], whey [43], *Klebsiella oxytoca* cultivation broth [38], and *Chlorella* sp. suspension [44].

The objective of this study was to assess the efficacy of air sparging as a technique for improvement of permeate flux during microfiltration of *Bacillus velezensis* IP22 cultivation broth, by using response surface methodology for modeling of the microfiltration. Further aim of the study was to maximize permeation flux while simultaneously minimizing specific energy consumption as a two-objective problem. For the optimization two approaches were analyzed, desirability function method and genetic algorithm. 

## 2. Materials and Methods

### 2.1. Production of Bacillus velezensis Cultivation Broth

*Bacillus velezensis* IP22, the strain used as an active component of microbial biopesticide, was isolated from fresh cheese and previously identified using 16S rRNA gene sequencing [11]. Preparation of inoculum, i.e., a sufficient amount of a liquid pure culture, was performed by transferring *Bacillus velezensis* IP22 biomass to Erlenmayer flasks containing sterile liquid medium—nutrient broth (HiMedia, Mumbai, India), followed by cultivation on a laboratory shaker at 28 °C, 150 rpm and under spontaneous aeration for 48 h. The obtained inoculum was used to inoculate the medium in the bioreactor (Biostat^®^ Aplus, Sartorius AG, Göttingen, Germany), while inoculum volume corresponded to 10% (*v*/*v*) of the bioreactor working volume (2 L). Cultivation medium was previously optimized for production of *Bacillus velezensis* IP22 biopesticide [11,12] and contained glycerol (10 g∙L^−1^), yeast extract (3 g∙L^−1^), (NH_4_)_2_SO_4_ (3 g∙L^−1^), K_2_HPO_4_ (1 g∙L^−1^), and MgSO_4_·7H_2_O (0.3 g∙L^−1^), while medium pH value was set to 7.0 ± 0.2. Bioreactor and medium sterilization was performed by autoclaving at 121 °C and 2.1 bar for 20 min. Cultivation of *Bacillus velezensis* IP22 in the bioreactor was carried out at 28 °C, with agitation rate of 250 rpm using Rusthone turbine with three impellers and with aeration rate of 2 L∙min^−1^ using sterile air and ring sparger for gas distribution. The obtained cultivation broth of *Bacillus velezensis* IP22 after 96 h of cultivation in the bioreactor was used as a feed mixture for microfiltration experiments.

In order to assess separation efficiency and viability of *Bacillus velezensis* cells, a standard plate count method was used, where dilutions of the cultivation broth (feed mixture), retentate, and permeate were prepared and used for inoculation of nutrient agar (Himedia, Mumbai, India). Petri dishes were incubated at 28 °C during 72 h, followed by colony enumeration. Cell concentration was also determined by measuring absorbance (optical density—OD_600_) of the cultivation broth, retentate, and permeate at a wavelength of 600 nm (UV 1800, Shimadzu, Kyoto, Japan). Biomass dry weight determination was performed as follows: biomass pellets obtained after centrifugation of cultivation broth and retentate samples (20 mL) were resuspended using 5 mL of distilled water and dried (105 °C) until reaching a constant weight. Centrifugation was performed at 11,000 rpm for 10 min (Rotina 1080R, Hettich, Kirchlengern, Germany) with sufficient driving force for sedimentation of intact cells, but insufficient for sedimentation of cell debris. The concentration of *Bacillus velezensis* (g∙L^−1^) was calculated using biomass dry weight and the volume of cultivation broth, retentate, or permeate sample (20 mL) which was centrifuged.

### 2.2. Microfiltration Experimental Setup

Experiments of *Bacillus velezensis* IP22 cultivation broth microfiltration were conducted using the previously described apparatus [25]. All microfiltration experiments were performed at 25 °C and with recirculation of retentate and permeate to maintain a constant volume of the feed mixture. The applied ceramic membrane (Tami Deutschland, Hermsdorf, Germany) had the following characteristics: length 250 mm, inner diameter 6 mm, outer diameter 10 mm, pore size 200 nm, and specific surface area 0.00433 m^2^ (designated as A in the Equations (1) and (2)). The pressurized air was introduced into the feed flow channel through the three-way valve without the diffusor. The air flow rate was measured by the mass flow controller type EL-FLOW F-201AV, with an accuracy of ±0.5% (Bronkhorst, Ruurlo, Netherlands). During microfiltration, the time (t) necessary to collect 20 mL (V) of permeate was measured and permeate flux value (J, L∙m^−2^·h^−1^) was calculated using the Equation (1):

(1) $$J=\frac{V}{A\cdot t}$$

Specific energy consumption per m^3^ of permeate (E, kW·h∙m^−3^) is equal to the ratio of sum of hydraulic and pneumatic powers to the permeate flow rate and it was calculated according to Equation (2):

(2) $$E=\frac{Q_{L}\cdot \left(P_{S}-P_{D}\right)+\frac{\gamma }{\gamma -1}\cdot P_{D}\cdot Q_{G,D}\cdot \left[{\left(\frac{P_{S}}{P_{D}}\right)}^{\frac{\gamma -1}{\gamma }}-1\right]}{J\cdot A}$$

where Q_L_ (m^3^∙h^−1^) is feed flow rate, P_S_ (Pa) is pressure at the beginning of the membrane module, P_D_ (Pa) is pressure at the end of the membrane module, Q_G,D_ (m^3^∙h^−1^) is air flow rate at the pressure at the end of the membrane module (P_D_), γ is the specific heat ratio for air (1.4), J (m^3^∙m^−2^·h^−1^) is permeate flux, and A (m^2^) is the specific membrane area.

### 2.3. Experimental Data Analysis—Modeling and Optimization

The Box–Behnken’s experimental plan (Table 1) with three independent variables (factors) varied at three levels was applied to investigate the effects of transmembrane pressure (TMP: 0.2–1 bar), superficial feed velocity (V_L_: 0.43–1.30 m∙s^−1^) and superficial air velocity (V_G_: 0.0–0.4 m∙s^−1^) to the following dependent variables (responses): steady state permeate flux (J, L∙m^−2^·h^−1^) and specific energy consumption (E, kW·h∙m^−3^). The obtained experimental results were fitted using the second-degree polynomial equation to obtain models for steady state permeate flux and specific energy consumption. The experimental data were analyzed using the factorial ANOVA (analysis of variance). The obtained *p*-values were used to assess statistical significance of the models’ coefficients and both models themselves, while quality of the experimental data fitting was estimated using the Lack-of-fit, Pure error, and R^2^ (coefficient of determination) values. The coefficient of determination value can be interpreted as the proportion of variability around the mean for the dependent variable, which can be accounted for by the model. It normally ranges from 0 to 1. Ideally, the value 1 means the perfect fit. A selected second-order polynomial model cannot fit perfectly the measured values, due to measurement errors or relationships between factors and responses that cannot be described by the selected model. Actually, this fact results in deviations of predicted values from the measured ones, i.e., so-called residual values exist at the design points. In the experimental designs, where some runs are replicated, such as the Box–Behnken’s experimental plan, ANOVA table will also include a lack-of-fit test. The statistical test based on partitioning the residual error sum of squares into two components: lack-of-fit sum of squares (associated with variation due to factors other than measurement error) and pure error sum of squares (associated with random variation caused by measurement error) is used to assess adequacy of the model. In fact, it is used to describe the functional relationships between the experimental factors and the responses. Low *p*-value for the lack-of-fit in the ANOVA table means that the analyzed model does not fit the experimental data adequately. All statistical analyses were performed at the significance level of 95% using the Statistica software (v. 13.5, Dell, Round Rock, TX, USA). 

The polynomial RSM models are usually used for the optimization by the desirability function approach [45]. One of many engineering optimization techniques is multi-objective genetic algorithm (GA) that represents a guided random search method. It is suitable for solving multi-objective optimization problems, capable of exploring the diverse regions of the solution space. The multi-objective optimization by the GA was performed by a non-dominated sorting genetic algorithm II (NSGA-II) [46], which generates a set of non-dominated Pareto optimal solutions. The plot of the Pareto front was drawn between the two objective functions: steady-state permeate flux (J) and specific energy consumption (E). The optimization problem studied is represented mathematically by Equation (3):

(3) $$\begin{array}{c} \max\;J\;\left(\mathrm{TMP},{\;V}_{L},{\;V}_{G}\right),\;\min E\;\left(\mathrm{TMP},{\;V}_{L},{\;V}_{G}\right)\\ \mathrm{subject}\;\mathrm{to}\;\mathrm{bound}\;\mathrm{constraints}\{\begin{array}{c} 0.2\;bar\;\leq TMP\;\leq 1\;bar\\ 0.43\;m\cdot s^{-1}\leq V_{L}\leq 1.30\;m\cdot s^{-1}\\ 0.0\;m\cdot s^{-1}\leq \;V_{G}\leq 0.4\;m\cdot s^{-1} \end{array} \end{array}$$

The Design-Expert software v. 8.1 (Stat-Ease, Inc., Minneapolis, MN, USA) was used for generating the polynomial RSM models and optimization by the desirability function approach, while for the GA optimization Matlab software (R2015b, MathWorks, Natick, MA, USA) was used.

## 3. Results

### 3.1. Modeling of Gas Sparging-Assisted Microfiltration of Bacillus velezensis IP22 Cultivation Broth

Microfiltration experiments were performed according to the Box–Behnken’s experimental plan (Table 1), where the effects of microfiltration operational conditions (transmembrane pressure, TMP—*X_1_*, superficial feed velocity, V_L_—*X_2_*, and superficial air velocity, V_G_—*X_3_*) to steady state permeate flux (J) and specific energy consumption (E) were investigated. Combinations of experimental factors (actual values of variables and coded values of variables in parentheses) and values of responses are summarized in Table 1. As the Box–Behnken’s experimental plan was defined for three independent variables varied at three levels, the coded values of independent variables represent the equally distant varied levels of the independent variables’ values (−1, 0, and 1).

The obtained experimental data were fitted using the second-degree polynomial equation to obtain models describing the effects of the aforementioned operational parameters to microfiltration performance. The linear (*b_1_*, *b_2_*, *b_3_*), quadratic (*b_11_*, *b_22_*, *b_33_*), and interaction (*b_12_*, *b_13_*, *b_23_*) model coefficients in terms of coded and actual variables’ values and the corresponding *p*-values are given in Table 2. Statistical significant coefficients, with *p*-values less than 0.05, are bolded in Table 2. The presented results have indicated statistical significance of linear effects of superficial feed velocity and superficial air velocity, quadratic effects of each independent variable and interaction effects of transmembrane pressure and superficial feed velocity, as well as superficial feed velocity and superficial air velocity, to steady state permeate flux. On the other hand, statistical significance for specific energy consumption was observed for the same effects as in the case of steady state permeate flux, with additional statistical significant effect of interaction between transmembrane pressure and superficial air velocity (Table 2).

ANOVA (analysis of variance) was performed to assess statistical significance of the obtained models for steady state permeate flux and specific energy consumption (Table 3). Based on the presented results, it could be concluded that the both second-degree mathematical models were statistically significant (with *p*-values less than 0.05, bolded in Table 3) and adequate in terms of quality of the experimental results fitting, with values of determination coefficient (R^2^) over 0.9. The ANOVA as given in Table 3 showed that coefficient of determination was 0.984 and 0.995, for second-degree polynomial models for permeate flux and specific energy consumption, respectively. That means that the permeate flux model could explain 98.4% of the variation in response, while in the case of specific energy consumption, the value is 99.5%, which indicated a superb fitness of the models. The high F values of the models (95.80 L∙m^−2^∙h^−1^ and 318.23 kW∙h∙m^−3^ for permeate flux and specific energy consumption, respectively), as well as non-significant lack-of-fit (0.22 for permeate flux and 0.26 for specific energy consumption) values showed that models were statistically significant. At the same time, small values of pure error (2.56 for permeate flux and 0.01 for specific energy consumption) indicated that variation caused by measurement error is insignificant. Therefore, these results are indicating that the selected regression model could be used to analyze trends of responses. Second-degree polynomial models could be successfully applied to describe the effects of transmembrane pressure, superficial feed velocity and superficial air velocity to steady state permeate flux and specific energy consumption during microfiltration of *Bacillus velezensis* IP22 cultivation broth aided with air sparging. 

**Figure 1:**
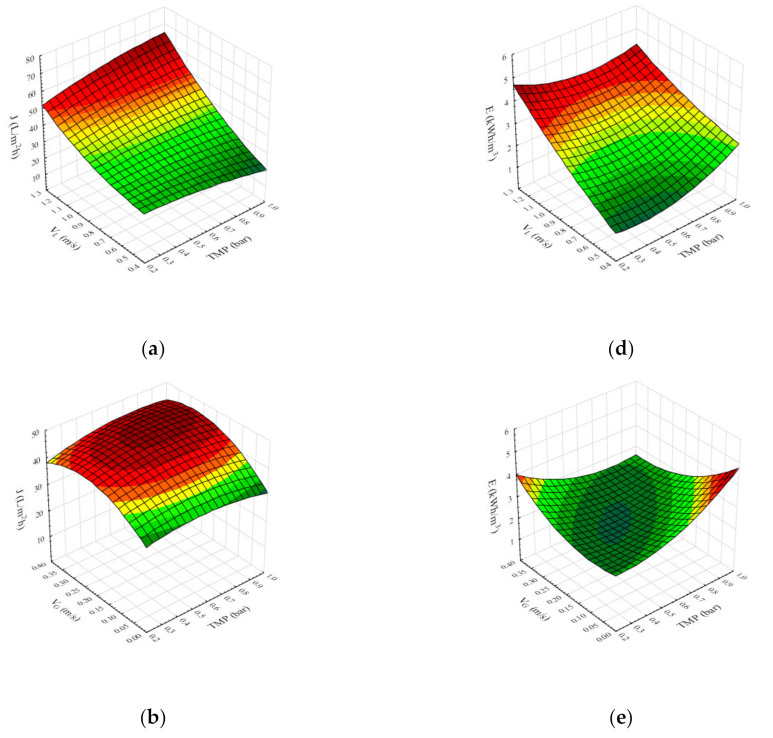

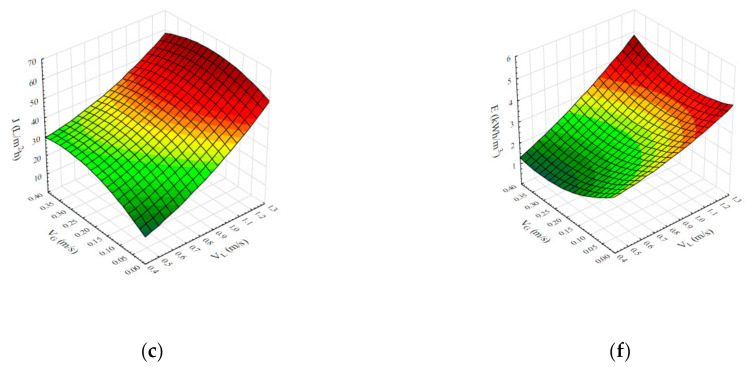
Response surface plots representing the regression models for steady state permeate flux (**a**–**c**) and specific energy consumption (**b**–**d**) during microfiltration of *Bacillus velezensis* IP22 cultivation broth aided with air sparging. The response surface plots represent the following effects of the independent variables to the aforementioned responses: (**a**) transmembrane pressure (TMP) and superficial feed velocity (V_L_) to permeate flux (J); (**b**) transmembrane pressure (TMP) and superficial air velocity (V_G_) to permeate flux (J); (**c**) superficial feed velocity (V_L_) and superficial air velocity (V_G_) to permeate flux (J); (**d**) transmembrane pressure (TMP) and superficial feed velocity (V_L_) to specific energy consumption (E); (**e**) transmembrane pressure (TMP) and superficial air velocity (V_G_) to specific energy consumption (E); (**f**) superficial feed velocity (V_L_) and superficial air velocity (V_G_) to specific energy consumption (E).

Furthermore, response surface plots were generated to better understand interactions of independent variables—operational conditions (transmembrane pressure, superficial feed velocity, and superficial air velocity)—to the selected microfiltration responses—steady-state permeate flux and specific energy consumption. The response surface plots (Figure 1) represent the effects of two independent variables to one response, while the value of the third independent variable was set to the mean value of the examined range of values. 

As can be seen in Figure 1a, high values of steady-state permeate flux were obtained at the highest applied values of superficial feed velocity across the whole range of transmembrane pressure values, with the highest value of permeate flux obtained at the highest value of transmembrane pressure applied. A similar effect could be observed when it comes to interaction of transmembrane pressure and superficial air velocity, with slight decrease of permeate flux at the highest value of superficial air velocity (Figure 1b). Furthermore, interaction of superficial feed velocity and superficial air velocity has showed that the highest value of steady state permeate flux was achieved at the highest value of superficial feed velocity across the whole range of superficial air velocity values (Figure 1c). 

On the other hand, the increase of specific energy consumption could be observed with the increase of superficial feed velocity, while the lowest value of specific energy consumption was obtained at the lowest value of transmembrane pressure (Figure 1d). The lowest value of specific energy consumption was achieved by applying middle range values of transmembrane pressure and superficial air velocity (Figure 1e). Interaction of superficial feed velocity and superficial air velocity has resulted in the lowest value of specific energy consumption at the lowest value of superficial feed velocity and the highest value of superficial air velocity (Figure 1f).

The analyses of cell viability and concentration have showed that *Bacillus velezensis* cells have been completely retained by the membrane. Biomass concentration of *Bacillus velezensis* in the fresh cultivation broth was 0.39 g∙L^−1^, while after the filtration experiments biomass concentration in the retentate was 0.36 g∙L^−1^. Spectrophotometric measurements (OD_600_) also confirmed this fact, and the absorbance values were 0.82 for the fresh cultivation broth and 0.77 for the retentate sample after microfiltration experiments. The results of the standard plate count method correspondingly suggested that the cells retained by the membrane did not suffer either significant change in the cell concentration nor decrease in the cell viability due to shear disintegration by pumping or mixer effects.

### 3.2. Optimization of Gas Sparging-Assisted Microfiltration of Bacillus velezensis IP22 Cultivation Broth

Optimization of operational conditions during air sparging-assisted microfiltration of *Bacillus velezensis* IP22 cultivation broth was performed using the desirability function approach. Optimization was aimed at maximizing steady state permeate flux and minimizing specific energy consumption. This method combines multiple responses into one response called the desirability function. The selected responses are transformed to an individual desirability values in range from 0 to 1. The overall desirability of the process is computed as a geometric mean of the individual desirability functions [24]. From the optimization results of the desirability function approach (Table 4) it can be concluded that the optimal results in terms of steady state permeate flux and specific energy consumption were obtained at transmembrane pressure value of 0.68 bar, superficial feed velocity of 0.96 m∙s^−1^ and superficial air velocity of 0.25 m∙s^−1^. The aforementioned optimized values of independent variables would result in predicted values of steady-state permeate flux of 48.57 L∙m^−2^·h^−1^ and specific energy consumption of 2.37 kW·h∙m^−3^. The optimized values of all three independent variables were close to the mean values of the tested range (0.2–1.0 bar for transmembrane pressure, 0.43–1.30 m∙s^−1^ for superficial feed velocity, and 0.0–0.4 m∙s^−1^ for the superficial air velocity). The obtained value of the desirability function was 0.62 (Table 4).

**Figure 2:**
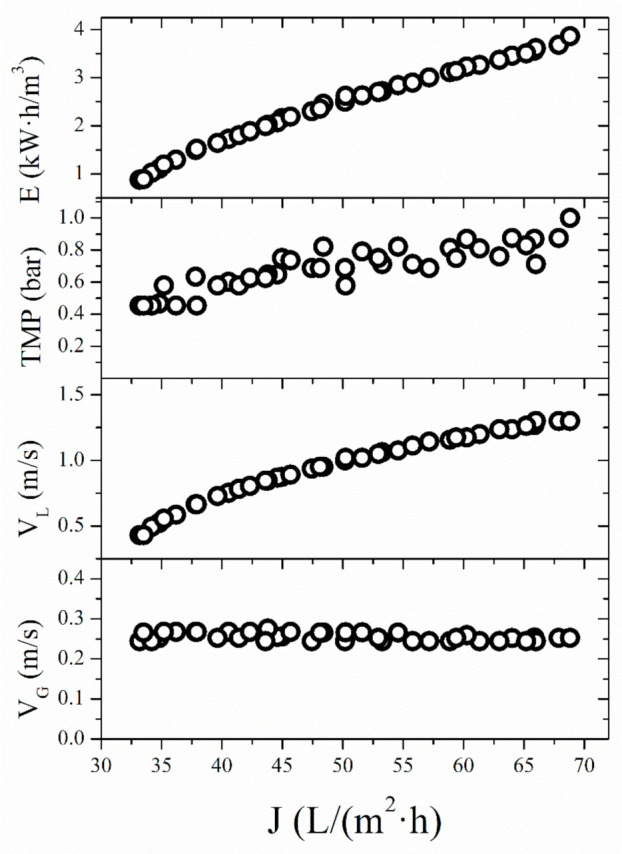
The Pareto front plot and decision space of the optimal solution set obtained from the multi-objective genetic algorithm optimization during microfiltration of *Bacillus velezensis* IP22 cultivation broth aided with air sparging.

The results of GA optimization are illustrated in Figure 2. The Pareto front reveals the conflicting relationship between steady-state permeate flux and specific energy consumption. The specific energy consumed per cubic meter of the permeate is equal to the ratio of sum of hydraulic and pneumatic powers to the permeate flow rate and is given by Equation (2). It can be reasoned that flux increase due to increased power consumption (both hydraulic and pneumatic) in some cases can result in lower values of specific energy consumption. This is the case when flux increase is sufficiently high to lower the ratio of energy consumption to permeate flux, given by Equation (2). The optimized values of transmembrane pressure had a distribution between 0.4 bar and 1.0 bar. Optimized value range of superficial feed velocity expanded on the whole range of the investigated experimental values, while on the other side, optimized value of superficial air velocity had a narrow distribution around 0.25 m∙s^−1^. As for the objective functions, their ranges were between 1 kW·h∙m^−3^ and 4 kW·h∙m^−3^ for specific energy consumption, and between 33 L∙m^−2^·h^−1^ and 70 L∙m^−2^·h^−1^ for steady-state permeate flux.

## 4. Discussion

### 4.1. The Effects of Operational Conditions on Steady State Permeate Flux during Air Sparging-Assisted Microfiltration of Bacillus velezensis IP22 Cultivation Broth

As it was previously stated, the obtained second-degree models for steady-state permeate flux and specific energy consumption during microfiltration of *Bacillus velezensis* IP22 cultivation broth aided with air sparging have proven to be statistically significant and appropriate for fitting of the obtained microfiltration experimental data. Furthermore, response surface plots were generated to better understand interactions of operational conditions (transmembrane pressure, superficial feed velocity, and superficial air velocity) to the selected responses (Figure 1).

The increase in steady-state permeate flux values was observed with the increase of superficial feed velocity value for all values of transmembrane pressure in the examined range (Figure 1a). Maximal permeate flux values are achieved in the region of the higher feed velocity values although this region corresponds to bubbly flow pattern, which is commonly associated with lower flux augmentations in the literature [33,36,47]. For the superficial feed velocity values of 0.43, 0.86, and 1.30 m∙s^−1^, the corresponding Reynolds’ number values are 1990, 3980, and 5971, respectively. Therefore, at the higher values of feed velocity turbulent flow exists even without gas sparging. The filtration cake thickness has reduced by increasing the superficial feed velocity, so the cake resistance to the flux flow was smaller and consequently the permeate flux value was higher. Due to the variation of bacterial cell arrangement in the cake layer, the permeate flux increase at the lower values of transmembrane pressure is less significant compared to the higher values of transmembrane pressure [48]. As superficial feed velocity increased from 0.43 to 1.30 m∙s^−1^, the permeation flux increased ~180% at the transmembrane pressure of 1 bar, while the increase of ~67% was observed in the same range of superficial feed velocity at the transmembrane pressure of 0.2 bar (Figure 1a). From the beginning of microfiltration at higher values of transmembrane pressure, the cells deposit randomly, as the cake formation is dominated by the permeation flow perpendicular to the membrane surface. On the other hand, with microfiltration progression permeation flux decreases, so the bacterial cells are arranged by the feed flow parallel to the membrane surface. The increase in superficial feed velocity results in cake thickness reduction, so the cell layer arranged by the feed flow is carried away, resulting in the reduced cake resistance, i.e., the influence of increase in superficial feed velocity is more pronounced at higher values of transmembrane pressure. Conversely, when lower values of transmembrane pressure are applied, the bacterial cell layer arrangement by the feed flow occurs earlier in the microfiltration process due to the low permeation fluxes [27,48], so at steady state, the increase of superficial feed velocity is not as effective in reducing cake resistance as for the higher values of transmembrane pressure. The influence of increase in transmembrane pressure on the steady state permeate flux is less manifested and to some extent ambiguous (puzzling) (Figure 1a). At the higher values of superficial feed velocity, increase of transmembrane pressure results in a moderate increase of steady state flux—approximately 24%. Increase of superficial feed velocity in membrane channel creates turbulence, which reduces the cake layer resistance by changing the fluid flow field and by increasing particle back transport through turbulent diffusion [49]. On the other hand, reduction in steady state permeate flux (~38%) with the increase of transmembrane pressure is observed at the lower values of superficial feed velocity (Figure 1a). The justification for this occurrence could be found in the shear-induced arrangement of *Bacillus velezensis* rod-shaped cells, that results in formation of the brick-like structure at the cake surface [20,50]. In the literature, similar results are reported for cross-flow microfiltration of other rod-like particles such as graphene oxide (16) or microorganisms with rod-shaped cells: *Pseudomonas* sp. [51], *Bacillus coagulans* [20], and *Escherichia coli* [52]. It seems that in the region of lower feed velocity values, turbulence caused by both air and feed flows is not sufficient to increase the steady state permeate flux by increasing transmembrane pressure. On the other hand, it is reported that air sparging might compress filtration cake to a more compact structure [33,34,40], so the cake resistance is higher as the cake is compacted more by raising transmembrane pressure and thus permeate flux value declines.

The effects of transmembrane pressure and superficial air velocity on the steady state permeate flux are given in Figure 1b. Increase of superficial air velocity up to the range of 0.25 m∙s^−1^ to 0.30 m∙s^−1^ resulted in increase of permeate flux at all transmembrane pressure values. However, further increase of superficial air velocity led to a modest permeate flux decline at lower values of transmembrane pressure. Similar results have been reported for air sparged microfiltration of yeast [34] and clay suspension [32]. In the case of clay suspension, it is stated that the maximal permeate flux was obtained at higher values of superficial air velocity indicating that filtration cake properties are greatly affected by the air sparging [32]. The influence of increase in superficial air velocity is more obvious at the higher values of transmembrane pressure because the membrane fouling was more severe compared to the lower values of transmembrane pressure. Similar results were reported for whey air sparging-assisted microfiltration [43]. As superficial air velocity value increased from 0.00 to 0.40 m∙s^−1^, the permeation flux has risen for around 45% at 1.0 bar, while its increase of ~23% has been observed in the same range of superficial air velocity values under transmembrane pressure of 0.2 bar (Figure 1b). As previously noted, another reason for this behavior can be found in the fact that air sparging might compress filtration cake to a more compact structure [40]. Hwang and Hsu [34] reported that during yeast air sparged microfiltration the cake properties were mainly determined by the yeast cells. The influence of cell shape on the cake structure is even more prominent for *Bacillus velezensis* rod-shaped cells compared to the oval yeast cells, as they tend to orientate parallel to feed flow [48,50]. In addition, an increase in filtration pressure leads to formation of cake with the higher resistance, so no influence of transmembrane pressure on the permeate flux was noted in the situation without air sparging. On the other hand, in the region of the highest values of superficial air velocity (the highest turbulence), ~14% increase in permeate flux value was achieved with an increase of transmembrane pressure (Figure 1b). As Figure 1b was drawn for the third factor (superficial feed velocity) set to its medium value (0.86 m/s) from the examined range, it is reasonable to assume that the higher values of superficial feed velocity would result in moderate rise of permeate flux with an increase of the transmembrane pressure due to improved turbulence in the membrane channel.

Figure 1c shows the simultaneous influence of superficial feed and air velocities on the steady state permeate flux. Increase of superficial air velocity value up to 0.40 m∙s^−1^ at the higher values of superficial feed velocity (1.30 m∙s^−1^) did not show significant effect to permeate flux, as the flux value has risen for ~6%. Substantial flux improvement was not achieved in these operational conditions as the high superficial feed velocity corresponds to turbulent regime (Reynolds’ number above 5900). Therefore, the air injection was not efficient enough to make the flow more turbulent [43]. On the other hand, in the lower value range of superficial feed velocity, increase of superficial air velocity resulted in larger increase of permeate flux. For example, at superficial feed velocity of 0.43 m∙s^−1^ increase of superficial air velocity up to 0.40 m∙s^−1^ resulted in increase of permeate flux value of 78% in the slug gas–liquid flow regime (Figure 1c). As reported in the literature, slug flow pattern induces enough turbulence to hinder cake formation [33,36,43,47]. At low values of superficial feed velocity (0.43 m∙s^−1^), a plateau in permeate flux values was observed during increase of superficial air velocity values from 0.25 m∙s^−1^ to 0.40 m∙s^−1^. This plateau became shorter with the increasing value of superficial feed velocity, while for the highest values of superficial feed and air velocities a decrease in permeate flux was observed. Air sparging reduces external membrane fouling by reducing filtration cake. The cake acts as a self-rejective dynamic membrane and protects membrane from internal fouling. Synergistic effect of both superficial feed and air velocities can cause the cake layer to become too thin and allow smaller components of the cultivation broth to penetrate into the membrane pores and thus reduce permeation flux [47]. Predictably, without air sparging, the permeate flux increased with the increase of superficial feed velocity as the higher wall shear stress reduced the cake formation. Nevertheless, the permeate flux improvement achieved by increasing superficial feed velocity is less significant combined with the higher values of superficial air velocity due to the increased level of turbulence. In the situation without air sparging, when superficial feed velocity value increased from 0.43 m∙s^−1^ to 1.30 m∙s^−1^, the permeation flux value rose for 200%, while it increased for ~80% in the same value range of superficial feed velocity, but under air sparging with superficial air velocity of 0.40 m∙s^−1^. 

The results of air sparged microfiltration of the *Bacillus velezensis* IP22 cultivation broth suggest that significant steady state permeate flux improvements could be achieved by applying two-phase flow. The increase in permeate flux is a result of not only an increase in superficial feed velocity by the two-phase flow, but also by the air flow itself, which creates instabilities in the feed flow [36]. This fact is proven by comparing the permeate flux values for the experiments with and without air sparging by selecting values of superficial air and feed velocities to be adjusted to levy the same mean velocity in the membrane channel. In the experiment without air sparging (TMP = 0.6 bar, V_L_ = 0.60 m∙s^−1^ and V_G_ = 0.00 m∙s^−1^) steady-state permeate flux value was 21.5 L∙m^−2^·h^−1^. On the other hand, in the experiment with the two-phase flow (TMP = 0.6 bar, V_L_ = 0.43 m∙s^−1^ and V_G_ = 0.18 m∙s^−1^) the permeate flux value was 29.4 L∙m^−2^·h^−1^, which represents an increase of 37%. From the economic point of view, investigation of specific energy consumption is necessary for the selection of the appropriate operational conditions. In the case of these two experiments, specific energy consumption values were 1.2 kW·h∙m^−3^ and 2.5 kW·h∙m^−3^ for the experiments with and without air sparging, respectively, suggesting that introduction of air into the membrane channel reduced specific energy consumption by 108%.

### 4.2. The Effects of Operational Conditions on Specific Energy Consumption during Air Sparging-Assisted Microfiltration of Bacillus velezensis Cultivation Broth

The efficiency of air sparging during microfiltration of *Bacillus velezensis* IP22 cultivation broth was also determined by investigating specific energy consumption as the energy dissipated per permeate volume unit. The influences of microfiltration operational conditions on the specific energy consumption are given in Figure 1. 

The increase in specific energy consumption is observed with the increase of superficial feed velocity for the whole value range of transmembrane pressure (Figure 1d). As the pumping energy is directly proportional to the feed flow rate, an increase in superficial feed velocity results in higher energy consumption. On the other hand, the increase of permeate flux in this regime was not high enough to compensate increased energy demand as the specific energy consumption is related to the permeate flow. As the increase of permeate flux was more pronounced for the higher values of transmembrane pressure (Figure 1a), consequently energy consumption per permeate volume unit was moderately rising (74%) for these experimental conditions compared to the rise of 300% at lower values of transmembrane pressure (i.e., 0.2 bar). At the higher values of superficial feed velocity, increase in transmembrane pressure resulted in permeate flux increase, so the specific energy consumption decreased for 10% in the selected experimental range. On the other side, *Bacillus velezensis* cell orientation in combination with increase of transmembrane pressure results in filtration cake with higher resistance [20,50]. Therefore, for lower permeate flux values energy consumption per permeate volume unit increased for ~130%, from 1.0 kW·h∙m^−3^ to 2.3 kW·h∙m^−3^. 

The effects of transmembrane pressure and superficial air velocity on the specific energy consumption are given in Figure 1e. The concave surface shape indicates that minimal values of the specific energy consumption (approximately 2.0 kW·h∙m^−3^) were recorded for median values of transmembrane pressure and superficial air velocity. Increase of superficial air velocity up to a range of 0.20 m∙s^−1^ to 0.30 m∙s^−1^ resulted in minor decrease of specific energy consumption at lower (0.2 bar) and median (0.7 bar) values of transmembrane pressure, respectively. Further increase of superficial air velocity caused incline of the specific energy consumption for the aforementioned value range of transmembrane pressure. The steepest increase (58%) in specific energy consumption was recorded at transmembrane pressure of 0.2 bar. However, at transmembrane pressure values higher than 0.7 bar, the trend of specific energy consumption decline remained for the full value range of superficial air velocity. The steepest decline (84%) of the specific energy consumption was recorded at transmembrane pressure of 1.0 bar (Figure 1e). Improved turbulence by applying higher value of superficial air velocity (0.40 m∙s^−1^) resulted in specific energy demand decrease by 52% for transmembrane pressure value rise from 0.2 to 1.0 bar (Figure 1e). In contrast, in the case without gas sparging specific energy consumption has risen 92% with increase of transmembrane pressure. At these experimental conditions, without air sparging or at low values of superficial air velocity, the increase of transmembrane pressure did not result in permeate flux increase. The reason for this is probably marginal influence of air bubbles on filtration cake that compacts with an increase of filtration pressure [44]. The increase is due to the fact that to reach higher values of transmembrane pressure, a higher value of feed pressure is needed, thus the insignificant change of flux results in higher specific energy consumption.

Figure 1f shows the simultaneous influence of superficial feed and air velocities on the specific energy consumption. Specific energy consumption increased with the increase of superficial feed velocity across the whole value range of superficial air velocity, by means of energy input of feed pumping. Due to the significant increase of permeate flux without air sparging with increased value of superficial feed velocity, the rise of specific energy consumption was hindered, and it was ~66%. For maximal values of superficial air velocity, this increase was steeper (310%)—from 1.1 kW·h∙m^−3^ to 4.5 kW·h∙m^−3^. In contrast to the influence of superficial feed velocity, increase of superficial air velocity value up to ~0.25 m∙s^−1^ resulted in reduction of specific energy consumption from 2.4 kW·h∙m^−3^ to 1.1 kW·h∙m^−3^, i.e., 120%. For further increase in superficial air velocity, there were no significant changes in specific energy consumption at lower values of superficial feed velocity. On the other hand, for higher values of superficial feed velocity (1.30 m∙s^−1^) increase of superficial air velocity over 0.25 m∙s^−1^ resulted in slight increase of 12.5% in energy consumption per permeate volume unit. This behavior can be explained by the membrane internal fouling triggered by a significant cake reduction in the area of high values of both feed and air velocities.

### 4.3. Optimization of Operational Conditions for Air Sparging-Assisted Microfiltration of Bacillus velezensis Cultivation Broth

Optimization of the operational parameters during microfiltration of *Bacillus velezensis* IP22 cultivation broth was performed using two approaches: desirability function method and genetic algorithm. The optimization was aimed at maximization of steady state permeate flux and minimization of specific energy consumption, in order to achieve maximal efficiency and cost effectiveness of microfiltration as the downstream operation in production of *Bacillus velezensis*-based biopesticide. When it comes to the optimization results, it could be concluded that there exists an opposite nature of the optimization goals, i.e., the antagonistic relationship of the examined dependent variables—steady-state permeate flux and specific energy consumption. The optimized value of superficial air velocity correlates with the results obtained by applying the response surface methodology. When it comes to superficial feed velocity and transmembrane pressure, the optimized values of these two independent variables were closer to the maximal values of the tested ranges as these values favor the maximization of permeate flux, but also result in an increase in specific energy consumption. Therefore, the satisfactory optimization solution by the desirability function approach (with desirability function value of 0.62) was achieved with the optimized values of independent variables that are close to the mean values of the examined value ranges (Table 4).

The optimum prediction by the desirability function approach is in agreement with the Pareto front. Even though the Pareto front offers many optimal solutions, the problem of choosing a compromise solution still exists. Suitable solution can be selected by taking into account the desired optimization goal(s), but trade-offs among different objectives are necessary [53]. If the optimization goal was set at maximal value of steady-state permeate flux, higher values of superficial feed velocity should be applied, as well as higher values of transmembrane pressure. In this case, specific energy consumption value was higher. By contrast, when the goal was to achieve lower values of specific energy consumption, lower values of superficial feed velocity should be used. When it comes to superficial air velocity, in all optimal solutions given by the Pareto front (Figure 2) optimized values were around 0.25 m∙s^−1^.

## 5. Conclusions

This study was aimed at investigating the effect of air sparging as a technique to achieve higher microfiltration efficiency when it comes to separation of *Bacillus velezensis* IP22 biomass aimed to be used as microbial biopesticide, as well as at modeling and optimization of the microfiltration process. The obtained second-degree polynomial models for dependent variables (steady-state permeate flux and specific energy consumption) have proven to be statistically significant and appropriate for fitting of the microfiltration experimental data. Application of the response surface methodology approach for microfiltration modeling has revealed significant effects of interactions between the main microfiltration parameters (transmembrane pressure, superficial feed velocity, and superficial air velocity) to the selected responses. Optimization was performed using the desirability function method and genetic algorithm, and it was aimed at maximization of steady-state permeate flux and minimization of specific energy consumption, as a two-objective problem. Both methods have revealed antagonistic relationship between the selected responses, while the optimal results of the operational parameters were set near median values of the investigated value ranges. The results of this study have confirmed a significant potential of air sparging to be used as a technique of choice for improvement of microfiltration efficiency and cost effectiveness. With improvement of permeate flux as a result, application of this technique primarily results in reduced membrane fouling, which also cuts down the membrane cleaning frequency and consequently increases the microfiltration operational time between the cleanings. In this way, membrane life cycle is also prolonged, which directly affects overall downstream cost, together with reduction of energy consumption during microfiltration. Considering high costs of biotechnological production of microbial biopesticides and high share of downstream processing in the overall bioprocess cost, air sparging-assisted microfiltration should be further examined as a downstream operation for not only production of microbial biopesticides, but also for downstream processing of other bacterial cultivation broths.

## Figures and Tables

**Table 1:** Box–Behnken’s experimental plan for *Bacillus velezensis* IP22 cultivation broth microfiltration experiments with air sparging—factors and responses.

Experiment	Factors—Independent Variables			Responses—Dependent Variables	
	TMP (bar)	V_L_ (m∙s^−1^)	V_G_ (m∙s^−1^)	J (L∙m^−2^·h^−1^)	E (kW·h·m^−3^)
1	0.2 (−1)	0.43 (−1)	0.2 (0)	31.06	1.1
2	1.0 (1)	0.43 (−1)	0.2 (0)	22.95	2.3
3	0.2 (−1)	1.30 (1)	0.2 (0)	55.89	4.4
4	1.0 (1)	1.30 (1)	0.2 (0)	70.00	3.9
5	0.2 (−1)	0.87 (0)	0.0 (−1)	30.57	2.4
6	1.0 (1)	0.87 (0)	0.0 (−1)	29.00	4.7
7	0.2 (−1)	0.87 (0)	0.4 (1)	36.67	3.8
8	1.0 (1)	0.87 (0)	0.4 (1)	41.47	2.5
9	0.6 (0)	0.43 (−1)	0.0 (−1)	17.50	2.3
10	0.6 (0)	1.30 (1)	0.0 (−1)	53.87	4.0
11	0.6 (0)	0.43 (−1)	0.4 (1)	32.64	1.1
12	0.6 (0)	1.30 (1)	0.4 (1)	58.05	4.6
13	0.6 (0)	0.87 (0)	0.2 (0)	43.45	2.1
14	0.6 (0)	0.87 (0)	0.2 (0)	42.80	2.1
15	0.6 (0)	0.87 (0)	0.2 (0)	45.00	2.0

TMP—transmembrane pressure, V_L_—superficial feed velocity, V_G_—superficial air velocity, J—steady state permeate flux, E—specific energy consumption.

**Table 2:** Coefficients of regression models for steady state permeate flux and specific energy consumption for microfiltration of *Bacillus velezensis* IP22 cultivation broth aided with air sparging.

Effects	Steady State Permeate Flux (L∙m^−2^·h^−1^)			Specific Energy Consumption (kW·h·m^−3^)		
	Coefficient		*p*-Value	Coefficient		*p*-Value
	Actual	Coded		Actual	Coded	
Intercept						
*b_0_*	**20.25**	**43.56**	**0.0141**	**0.78**	**2.05**	**0.0302**
Linear						
*b_1_*	−10.54	1.12	0.1410	0.37	0.21	0.0009
*b_2_*	**−9.62**	**16.71**	**<0.0001**	**0.88**	**1.26**	**<0.0001**
*b_3_*	**107.98**	**4.75**	**0.0007**	**−5.40**	**−0.18**	**0.0021**
Quadratic						
*b_11_*	**−15.20**	**−2.43**	**0.0017**	**3.78**	**0.60**	**0.0002**
*b_22_*	**20.34**	**3.85**	**0.0096**	**1.42**	**0.27**	**<0.0001**
*b_33_*	**−172.28**	**−6.89**	**0.0295**	**16.98**	**0.68**	**0.0001**
Interaction						
*b_12_*	**31.90**	**5.55**	**0.0499**	**−2.45**	**−0.42**	**<0.0001**
*b_13_*	19.91	1.59	0.1400	**−11.25**	**−0.90**	**0.0018**
*b_23_*	**−31.51**	**−2.74**	**0.0008**	**5.17**	**0.45**	**<0.0001**

**Table 3:** ANOVA of regression models for steady state permeate flux and specific energy consumption for microfiltration of *Bacillus velezensis* IP22 cultivation broth aided with air sparging.

Source	Response	DF	SS	MS	F-Value	*p*-Value	R^2^
Model	J (L∙m^−2^·h^−1^)	9	2845.19	316.13	95.80	**0.000046**	0.984
	E (kW·h∙m^−3^)	9	21.06	2.34	318.23	**0.000002**	0.995
Residual	J (L∙m^−2^·h^−1^)	5	16.50	3.30			
	E (kW·h∙m^−3^)	5	0.04	0.01			
Lack-of-fit	J (L∙m^−2^·h^−1^)	3	13.94	4.65	3.64	0.22	
	E (kW·h∙m^−3^)	3	0.03	0.01	3.01	0.26	
Pure error	J (L∙m^−2^·h^−1^)	2	2.56	1.28			
	E (kW·h∙m^−3^)	2	0.01	0.00			
Total	J (L∙m^−2^·h^−1^)	14	2861.68				
	E (kW·h∙m^−3^)	14	21.10				

J—steady state permeate flux, E—specific energy consumption, DF—degree of freedom, SS—sum of squares, MS—mean squares, R^2^—coefficient of determination.

**Table 4:** Optimization results obtained by the desirability function approach during microfiltration of *Bacillus velezensis* IP22 cultivation broth aided with air sparging.

**Factors—independent variables**	**Goal**	**Optimized value**
Transmembrane pressure, TMP (bar)	in range	0.68
Superficial feed velocity, V_L_ (m∙s^−1^)	in range	0.96
Superficial air velocity, V_G_ (m∙s^−1^)	in range	0.25
**Responses—dependent variables**	**Goal**	**Predicted value**
Steady state permeate flux, J (L∙m^−2^·h^−1^)	maximize	48.57
Specific energy consumption, E (kW·h∙m^−3^)	minimize	2.37
**Desirability function**	0.62	

## Data Availability

Not applicable.
